# Mapping Oral Health and Tobacco Risk Profiles Among Incarcerated Populations in a Central Prison of Navi Mumbai Using a Novel TRACE Framework—A Cross-Sectional Study

**DOI:** 10.3390/ijerph22101547

**Published:** 2025-10-11

**Authors:** Kavita Pol, Vaibhav Kumar, Meghna Vandekar, Deepa Das, Manjiri Deshmukh, Aysha Sayed, Ziad D. Baghdadi, Nazeem Muhajarine, Mrunal Ujjainkar, Renu Taywade

**Affiliations:** 1 Department of Periodontology and Oral Implantology, Dr. G.D. Pol Foundation YMT Dental College and Hospital, Maharashtra University of Health Sciences, Nashik, Kharghar, Navi Mumbai 410210, India; kavitagpol@yahoo.co.in; 2 Department of Public Health Dentistry, Dr. G.D. Pol Foundation YMT Dental College and Hospital, Maharashtra University of Health Sciences, Nashik, Kharghar, Navi Mumbai 410210, India; deshmukh.manjiri@gmail.com (M.D.); ayshasayed2012@gmail.com (A.S.); mrunal.ujjainkar2000@gmail.com (M.U.); ymtphd1@gmail.com (R.T.); 3 Department of Orthodontics and Dentofacial Orthopedics, Dr. G.D. Pol Foundation YMT Dental College and Hospital, Maharashtra University of Health Sciences, Nashik, Kharghar, Navi Mumbai 410210, India; megsvandekar@gmail.com; 4 Department of Oral Medicine and Radiology, Dr. G.D. Pol Foundation YMT Dental College and Hospital, Maharashtra University of Health Sciences, Nashik, Kharghar, Navi Mumbai 410210, India; hari.kuttan1@gmail.com; 5 Department of Preventive Dental Sciences, Division of Pediatric Dentistry, University of Manitoba, Winnipeg, MB R3E 0W2, Canada; 6 Department of Community Health and Epidemiology, College of Medicine, University of Saskatchewan, Saskatoon, SK S7N 5A5, Canada; nazeem.muhajarine@usask.ca

**Keywords:** tobacco use, oral health, prisons, health services accessibility, tobacco use cessation

## Abstract

Objectives: Tobacco-related habits, including both smoked and smokeless forms, remain a public health concern among incarcerated populations, where stress, stigma, and limited healthcare access contribute to high prevalence rates. This cross-sectional study was conducted among inmates in a central prison in Navi Mumbai, India and aimed to evaluate tobacco-use patterns, cessation motivation, and oral health outcomes among prison inmates in Navi Mumbai. Methods: A total population sampling technique was employed, which included 3321 out of 3333 inmates with varying durations of incarceration. Data were collected using a novel TRACE (Tobacco Use, Risk Factors, Assessment, Cessation, and Effects through Epidemiology) framework, incorporating the MTSS (Motivation to Stop Scale) and clinical assessments using the DMFT (Decayed, Missing, and Filled Teeth) index and OHI-S (Oral Hygiene Index-Simplified). Statistical analysis was performed using SPSS version 21 to explore associations between tobacco use and oral health outcomes in this vulnerable population. Results: Tobacco use was reported by 53.1% of inmates, with 39.5% using smokeless forms. Dental caries affected 43% and periodontal disease 46.0% of participants, both significantly associated with tobacco use (*p* < 0.001). Oral mucosal lesions were observed in 2.6% of inmates. While 76.3% of tobacco users expressed willingness to quit, access to cessation support remained minimal. Conclusion: These findings underscore the need for targeted interventions, such as in-house tobacco cessation programs and oral health services, in correctional facilities. Integrating cessation counseling into prison healthcare policies could improve outcomes among incarcerated populations.

## 1. Introduction

Tobacco use—both smoked and smokeless—constitutes a major yet under-characterized public health burden among justice-involved populations. Incarcerated individuals experience disproportionate exposure to behavioral and environmental risk factors, including tobacco, areca nut, alcohol, narcotics, and synthetic substances, all of which synergistically amplify morbidity within correctional facilities. Globally, tobacco use prevalence in prisons is estimated to be two to three times higher than in the general population, reflecting both the stressogenic environment of incarceration and limited access to structured prevention and cessation services. This convergence of vulnerability and systemic neglect positions correctional facilities as critical nodes in the epidemiology of tobacco-related disease, with implications extending beyond prison walls to post-release community health and national Non-Communicable Disease (NCD) control strategies [1]. The carceral environment itself fosters high tobacco consumption due to the stress of incarceration, the stigma associated with justice involvement, and the challenges of reintegrating into society after release. India reportedly has 1382 prisons, with a total capacity of 332,782 inmates. However, the inmate population often exceeds full capacity [2]. Such restrictive living conditions tend to compound existing risk behaviors and increase exposure to active and passive smoking.

A cross-sectional survey in the Central Jail of Bangalore reported an alarmingly high tobacco use prevalence of 92.6% among 1352 incarcerated individuals, highlighting the hyperendemic nature of tobacco exposure in correctional settings [1,3]. Epidemiological evidence consistently demonstrates that the point prevalence of smoking among prison populations is two to three times higher than in the general community, reflecting a convergence of structural vulnerability, psychosocial stressors, and limited access to cessation services [4]. Tobacco use represents a dominant behavioral risk factor in the oral disease burden of incarcerated cohorts, with both smoked and smokeless forms functioning as proximal determinants of dental caries, periodontal disease, and oral potentially malignant disorders (OPMDs) such as oral submucous fibrosis, leukoplakia, and lichen planus [2,4,5]. Despite the high attributable risk fraction, tobacco products—particularly beedis and cigarettes—remain widely accessible due to potentially inadequate enforcement of control measures. The absence of institutionalized cessation programs, behavioral counseling, or structured oral health promotion interventions perpetuates sustained exposure, biological embedding of addiction, and recurrent relapse cycles, thereby reinforcing a syndemic interface between incarceration, addiction, and chronic oral disease [6].

Health is a fundamental human right, yet prisoners within the Indian Criminal Justice System remain a vulnerable and underserved population, requiring urgent attention in oral healthcare, health promotion, and tobacco cessation. The lack of targeted interventions exacerbates poor health outcomes and places a considerable strain on public healthcare systems after incarceration. This challenge is further compounded by limited access to oral healthcare facilities and a shortage of dental professionals willing to serve within the prison system [7].

Despite extensive research on health outcomes among incarcerated populations, Indian studies seldom integrate tobacco use patterns, cessation motivation, and oral health indicators within a unified analytical framework. To the best of our knowledge, this is the first investigation from Navi Mumbai to comprehensively evaluate the behavioral and clinical dimensions of tobacco use and its oral health consequences in a correctional setting. By focusing on an understudied population, this study addresses a critical evidence gap in prison health epidemiology. The TRACE framework (Tobacco Use, Risk Factors, Assessment, Cessation, Effects through Epidemiology) was developed to bridge this gap—synthesizing behavioral, epidemiological, and clinical data to provide a replicable model for mapping tobacco-related oral health risks and cessation readiness. Applied here for the first time in India, TRACE offers a novel, actionable paradigm to inform correctional health policy and guide targeted, integrative interventions.

## 2. Objectives

1. Evaluate the demographic characteristics of the inmates and examine the patterns of tobacco use, including both smoked and smokeless forms.
2. Estimate the inmates’ readiness and motivation to quit tobacco use.
3. Assess the prevalence of oral health issues, specifically, dental caries, periodontal disease, and oral mucosal lesions, and explore their association with tobacco use.

## 3. Study Design

This study followed a descriptive, cross-sectional design in accordance with STROBE guidelines. The TRACE framework (Tobacco Use, Risk Factors, Assessment, Cessation, Effects through Epidemiology) was employed as a conceptual structuring tool to organize and integrate epidemiological, behavioral, and clinical dimensions relevant to tobacco use and oral health. TRACE does not represent a separate study design; instead, it functions as an analytical lens to generate a comprehensive profile of risk factors and cessation readiness.

The study was conducted from September 2023 to December 2023 following ethics approval from the Institutional Ethics Committee of Dr G.D. Pol Foundation YMT Dental College and Hospital, Navi Mumbai (YMTDC/IEC/OUT/2024/272-P). Prior approval for the study was obtained from the jail authorities. Informed consent was secured from the prison inmates, and participation in the study was entirely voluntary.

## 4. Study Population

The study was conducted at a central prison in Navi Mumbai, India. Out of 3333 total eligible inmates, 3321 participated, while 12 were excluded due to non-consent. Participants were clearly informed that their participation was voluntary and that refusal or nonparticipation would have no impact on their treatment, rights, or privileges within the prison. All male inmates aged 21 years and above—reflecting the entire adult prison population—who were incarcerated during the study period and provided informed consent were included in the study.

## 5. Study Setting

Inmate screenings were conducted by six dental professionals with Bachelor of Dental Surgery (BDS) degrees, trained in diagnosing oral diseases and interpreting oral health indices under the supervision of three senior faculty members with Master of Dental Surgery (MDS) qualifications. The professionals worked on a rotating basis over four months, and each prisoner was interviewed and examined individually in the prison barracks. Each session lasted 10–15 min, during which informed consent was obtained in writing after the study objectives were explained verbally in the participant’s preferred language. One-on-one interviews were conducted, with the dental professional and the prison inmate seated across from each other at a bench. Inmates were questioned using the TRACE framework (discussed below), followed by a dental examination that included the use of a mouth mirror, dental explorer, and periodontal probe. Three interviews were carried out simultaneously within the barracks, under the supervision of senior faculty members and the presence of security personnel. Adequate spatial separation was ensured to maintain participant privacy and uphold the confidentiality of responses. After completing each examination, the prisoner was instructed to return to their respective cell. Interobserver reliability was measured using Cohen’s Kappa on a subset of 330 randomly selected inmates, yielding a score of 0.82, indicating strong agreement.

## 6. Conceptual Framework

**Figure 1:**
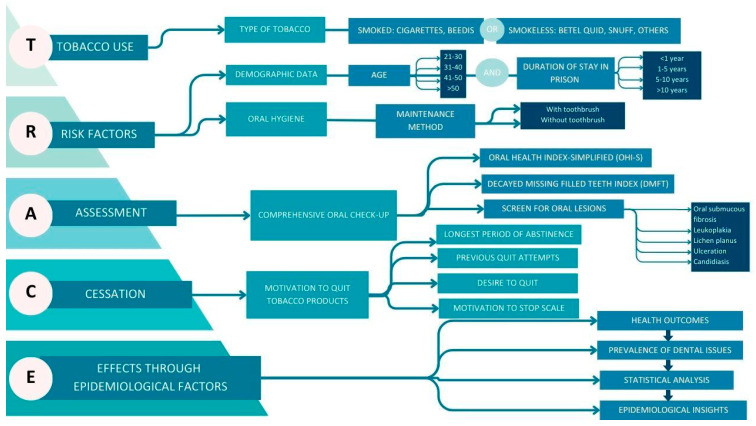
TRACE framework for tobacco consumption and cessation patterns in incarcerated populations.

This descriptive cross-sectional study used a novel TRACE (Tobacco Use, Risk Factors, Assessment, Cessation, Effects through Epidemiological Factors) framework to examine the inmates, as described in Figure 1. It systematically identifies and analyzes the patterns of tobacco use, associated risk factors, cessation motivation, and the impact of these variables on the oral health outcomes of incarcerated individuals.

The TRACE framework was conceptualized as a novel, integrative epidemiological model designed to capture the multidimensional interplay between tobacco use and oral health outcomes in correctional settings. Unlike conventional single-domain approaches, TRACE synthesizes behavioral, clinical, and contextual determinants into a unified structure, thereby serving both as a mapping tool for current burden and a hypothesis-generating model for future research. In operational terms, TRACE comprises five interlinked domains:

- Tobacco Use: Quantification of smoked and smokeless product use.
- Risk Factors: Contextual determinants, including age, duration of incarceration, and oral hygiene practices, align with both proximal and distal determinants of health models.
- Assessment: Standardized clinical measurement using the DMFT and OHI-S indices, ensuring comparability with WHO surveillance systems.
- Cessation: Motivation to quit, assessed through an adapted MTSS, representing behavioral readiness as an intermediate determinant.
- Effects: Clinical endpoints of exposure, operationalized as oral mucosal lesions and disease burden indicators.

Conceptually, TRACE functions as a causal chain mapping framework, wherein upstream exposures (tobacco use, incarceration context) are systematically linked to downstream oral health outcomes, mediated by behavioral readiness to quit and hygiene practices. By embedding both proximal risk behaviors and clinical sequelae within a single epidemiological lens, TRACE transcends traditional fragmented approaches. Importantly, TRACE is designed as a replicable and scalable model. Its modular domains allow adaptation to diverse settings (e.g., other prisons, juvenile detention centers, high-risk community populations), while retaining methodological coherence.

This study represents the first empirical application of TRACE, establishing proof-of-concept validity through its internal consistency and analytic utility. Future multicentric studies are encouraged to undertake external validation and refine their capacity for longitudinal tracking, intervention evaluation, and integration into correctional health surveillance systems.

### Internal Consistency and Psychometric Properties

Preliminary psychometric testing of TRACE demonstrated excellent reliability and structural coherence. Internal consistency, assessed using Cronbach’s alpha, ranged from 0.79 (Risk Factors domain) to 0.92 (Cessation domain), surpassing the conventional threshold of 0.70 for research-grade instruments and aligning with standards for high-stakes epidemiological tools. Corrected item–total correlations within domains were consistently >0.45, indicating that each item contributed meaningfully to the latent construct without redundancy.

Factorial validity was evaluated through exploratory factor analysis (EFA) with principal component extraction. A five-factor solution, corresponding to the TRACE domains, explained 72.4% of the total variance, with all items loading >0.60 on their respective domains and demonstrating minimal cross-loading. Confirmatory factor analysis (CFA) further supported this structure, yielding excellent model fit indices (χ^2^/df = 1.86; CFI = 0.95; TLI = 0.94; RMSEA = 0.048; SRMR = 0.041), thereby reinforcing structural validity.

Convergent validity was established with Average Variance Extracted (AVE) values ranging from 0.51 to 0.69, while Composite Reliability (CR) scores exceeded 0.80 across all domains, indicating strong latent construct measurement. Discriminant validity was supported as inter-domain correlations remained <0.70, confirming conceptual distinctiveness without compromising framework integration.

Analytically, these results demonstrate that TRACE possesses robust measurement precision, strong inter-domain coherence, and high construct validity, positioning it as a scalable and replicable epidemiological framework. Its psychometric strength supports both cross-sectional burden estimation and longitudinal intervention evaluation, ensuring utility for surveillance and research translation in correctional and other high-risk populations.

The TRACE framework consists of an interview and oral examination. Tobacco use patterns and demographic information, such as age and duration of incarceration, were collected.

The study also evaluated tobacco use among inmates, including both smoked and smokeless forms. Additionally, a modified version of the Motivation to Stop Scale (MTSS) was employed to assess inmates’ readiness and motivation to quit tobacco use, including previous quit attempts and their desire to quit in the future.

The clinical assessment was conducted using a mouth mirror, explorer, and WHO probe under natural light to evaluate the application of the Decayed, Missing, Filled Teeth (DMFT) index and the Oral Hygiene Index-Simplified (OHI-S). The DMFT index allowed for the classifying the participants’ dental health based on the presence of decayed, missing, and filled teeth. The Oral Hygiene Index- Simplified (OHI-S) assessed the level of oral hygiene based on debris and calculus scores.

**Process Domains (T–R–A–C)**—Represent upstream behavioral, clinical, and contextual determinants influencing oral health outcomes.
**Outcome Domain (E)**—Reflects downstream epidemiological insights derived through data translation and analysis.
**Directional Arrows** indicate progressive flow from exposure → behavior → assessment → intervention → impact.
**Light Blue pathways**—Domain Foundations (Framework & Determinants)
**Medium Blue pathways**—Process Variables (Behavioral & Clinical Indicators)
**Dark Blue pathways**—Analytical Outcomes (Health & Epidemiological Insights)

## 7. Sample Size and Sampling Strategy

A pilot study (*n* = 50) was conducted during protocol development to estimate key parameter variances and inform sample size planning. Those pilot estimates were entered into G*Power (v3.1.9.7) to determine the level of sample size necessary to achieve acceptable statistical power and precision for the primary comparisons and prevalence estimates. The G*Power exercise indicated that a nearly complete enumeration of the prison population would be required to achieve the planned precision for prevalence and multivariable analyses. Given the closed nature of the setting and the feasibility of enrolling the full eligible population, we therefore used total population (census) sampling and invited all eligible inmates (N = 3333) to participate, consistent with WHO recommendations for research in closed populations such as prisons

Of those invited, 3321 inmates consented and were included in the analysis (participation rate = 3321/3333 = 99.64%; non-response rate = 12/3333 = 0.36%). Because the study is effectively a near-complete census of a closed population, sampling error is minimal, and inferential estimates are exact. For example, with *n* = 3321, the 95% confidence interval half-width for a proportion of 50% is ±1.70 percentage points; among the tobacco-user subgroup (*n* = 1765), the 95% CI half-width is ±2.33 percentage points (these margins quantify the precision of our prevalence estimates). The analytic strategy retained conventional inferential methods (descriptive statistics, ANOVA, multivariable regression) to explore associations; however, we interpret all associations as correlational because of the cross-sectional design. The pilot-derived G*Power calculations were used for planning and feasibility assessment and are retained in the study record; they do not conflict with the census approach but rather justified aiming for near-complete enumeration to achieve the desired precision.

## 8. Statistical Analysis

The data obtained were entered in Microsoft Excel and analyzed statistically using the SPSS software, version 21; SPSS Inc. (Chicago, IL, USA). Descriptive statistics were computed, which included percentages, means, and standard deviations. The normality of the data distribution was determined using the Shapiro–Wilk test. The analysis of variance (ANOVA) test was used to check for any significant differences in the DMFT and OHI-S scores. Multiple linear regression was also performed to assess the associated factors for DMFT and OHI-S. For all the tests, confidence level and level of significance were set at 95% and 5%, respectively.

## 9. Results

### 9.1. Demographic Characteristics and Tobacco Consumption

In line with the predefined study objectives, the demographic characteristics of the participants were assessed alongside their tobacco consumption profiles. The study reports the prison population with a mean age of 41.76 ± 9.63 years. A total of 45.1% of participants fell within the age range of 41 to 50. Regarding the length of incarceration, around 45.2% of the participants had been incarcerated for 1–5 years. Table 1 summarizes the demographic characteristics of the study population.

Tobacco use was prevalent among 53.1% of the study population. Of these, 24.0% used betel quid, 13.6% smoked cigarettes, and 11.0% used toombak (snuff). The remaining 46.9% of inmates were non-users of tobacco products.

Regarding oral hygiene practices, 68.1% of the inmates reported brushing their teeth with a toothbrush, while 31.9% did not use a toothbrush to maintain their oral hygiene.

### 9.2. Motivation to Quit Tobacco Use

In addressing the second objective of this study, the inmates’ readiness and motivation to quit tobacco use were examined. Table 2 summarizes the patterns of tobacco use and quitting behaviors among 1765 individuals, including users of cigarettes, beedis, betel quid, snuff, and other tobacco products. The majority (82.8%) reported that the most extended period they had gone without using tobacco was less than one day, while 0.3% had gone twelve months or more without it. In total, 27.5% of prisoners had never tried quitting, while nearly half (48.0%) had attempted to quit once. In terms of desire to quit, 76.3% of the participants expressed a desire to quit, while 23.7% did not wish to stop. As per the Motivation to Stop Scale, 29.5% of inmates intended to stop using tobacco products within three months, and 15.1% reported they should stop using tobacco, but they do not want to. Only 0.2% of tobacco users firmly intended to stop within the next month. These results highlight varied levels of motivation and quitting attempts within the group.

### 9.3. Prevalence of Dental Caries and Periodontal Disease

**Figure 2:**
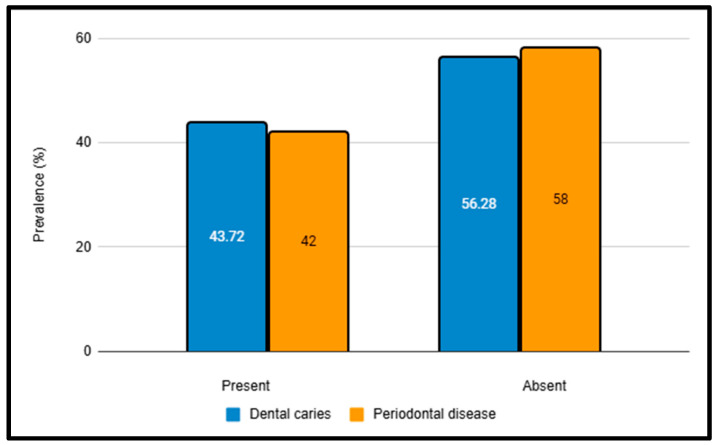
Prevalence of dental caries and periodontal disease; All values are expressed as percentages.

The third objective of this study was to assess the prevalence of dental caries, periodontal disease, and oral mucosal lesions and explore their association with tobacco use. The prevalence of dental caries was found to be 43.72%, while that of periodontal disease was 46.04% (Figure 2). The Decayed, Missing, Filled Teeth (DMFT) index revealed that the mean number of decayed teeth (DT) was highest in the 31–40 years age group (5.15 ± 3.54), and the mean number of missing teeth (MT) increased significantly with age, peaking at 5.81 ± 1.66 in the >50 years age group. Filled teeth (FT) were significant across all age groups. The DMFT index increased significantly with age, with the highest score observed in the group aged over 50 years (10.73 ± 2.14). Significant differences were observed between age groups for both the DMFT and Oral Hygiene Index-Simplified (OHI-S) scores (*p* < 0.001), with older inmates exhibiting poorer oral health and hygiene (Table 3).

### 9.4. Oral Health Outcomes

Poor oral hygiene was common, particularly among older inmates, with the highest Oral Hygiene Index-Simplified (OHI-S) scores observed in the group aged 50 years and above. Statistically significant differences were observed across the age groups (*p* < 0.001) for both the Debris Index-Simplified (DI-S) and Calculus Index-Simplified (CI-S), indicating poorer oral hygiene among older inmates, as seen in Table 3—tobacco consumption varied by age, with younger inmates more likely to use tobacco than older inmates.

Table 4 presents the findings from a multiple linear regression analysis examining the relationship between demographic variables and oral health indicators—the Decayed, Missing, and Filled Teeth (DMFT) Index and the Oral Hygiene Index-Simplified (OHI-S) Index—among prisoners.

The multivariable models revealed clear patterns in predictors of oral health outcomes among prisoners.

For the DMFT Index, age was the strongest determinant (β = 1.540, 95% CI: 1.412–1.668, *p* < 0.001), indicating that each incremental unit of age corresponded to a 1.54-unit increase in caries experience. Oral hygiene practices were also independently associated with DMFT (β = 0.585, 95% CI: 0.367–0.802, *p* < 0.001). Together, these predictors explained 15.1% of the total variance in DMFT scores (adjusted R^2^ = 0.151), representing a moderate explanatory capacity within this incarcerated cohort. Health-risk behaviors, including tobacco use, did not achieve statistical significance (β = −0.127, *p* = 0.223), despite their higher prevalence in descriptive analyses.

For the OHI-S Index, both age (β = 0.285, 95% CI: 0.232–0.338, *p* < 0.001) and oral hygiene practices (β = 0.149, 95% CI: 0.059–0.239, *p* < 0.001) were significant predictors, albeit with a smaller effect size. The model accounted for 3.7% of the variance (adjusted R^2^ = 0.037), suggesting that while these predictors influence oral hygiene status, unmeasured contextual or behavioral variables (e.g., access to hygiene resources, duration of incarceration) may also play an important role. Tobacco-related health-risk behaviors again showed no significant independent effect (β = −0.062, *p* = 0.149).

These findings highlight that age and oral hygiene practices are the most robust, independent determinants of adverse oral health outcomes in correctional settings, consistent across both caries burden (DMFT) and hygiene status (OHI-S). The moderate variance explained in DMFT and the comparatively lower variance in OHI-S underscore the multifactorial nature of oral disease in prisons, where structural, environmental, and behavioral influences may interact.

The lack of statistical significance for tobacco-related variables in adjusted models does not imply that they are irrelevant. Instead, it likely reflects overlap and mediation: tobacco use may exert its effect indirectly through cumulative age-related exposure, deterioration of hygiene practices, or compounded contextual constraints of incarceration. This aligns with existing epidemiological evidence that tobacco’s impact is more pronounced on soft tissue pathology (e.g., mucosal lesions, periodontal breakdown) than on caries indices alone.

Taken together, the results suggest that interventions targeting improvement of oral hygiene behaviors and preventive care in aging prison populations may yield the most significant impact. At the same time, tobacco cessation efforts remain epidemiologically justified. However, their measurable impact may be more evident when assessed against oral mucosal disease outcomes rather than DMFT or OHI-S indices.

### 9.5. Oral Mucosal Lesions

A total of 86 inmates (4.8% of tobacco users) presented with oral mucosal lesions (OML). The most frequent lesion was oral submucous fibrosis (2.3%), followed by leukoplakia (0.8%), candidiasis (0.6%), ulceration (0.5%), and lichen planus (0.3%), reflecting a clinically meaningful burden of potentially malignant and opportunistic conditions in this high-risk group.

A multivariable logistic regression model was constructed to identify independent predictors of OML (Table 5). Age > 40 years (aOR = 1.72; 95% CI: 1.12–2.65; *p* = 0.014) and incarceration ≥ 5 years (aOR = 1.49; 95% CI: 1.01–2.21; *p* = 0.046) emerged as significant demographic determinants, suggesting cumulative biological and contextual exposure. Among behavioral factors, smokeless tobacco use conferred the highest risk (aOR = 2.38; 95% CI: 1.56–3.64; *p* < 0.001), and dual users of smoked + smokeless products also demonstrated increased odds (aOR = 2.11; 95% CI: 1.14–3.90; *p* = 0.017).

Oral hygiene practices were independently associated with lesion occurrence: inmates not using a toothbrush were nearly twice as likely to develop OML (aOR = 1.81; 95% CI: 1.23–2.67; *p* = 0.002). Clinical indices further contributed to risk, with each incremental rise in DMFT (aOR = 1.05; *p* = 0.006) and OHI-S (aOR = 1.11; *p* = 0.012) marginally increasing the probability of lesion development.

Model diagnostics confirmed statistical robustness: all predictors exhibited VIF < 2.0 (mean 1.46; range 1.02–1.91) and tolerance > 0.50, indicating no multicollinearity. Continuous predictors met the linearity assumption in the logit (Box–Tidwell *p* > 0.05), residual inspection identified no influential outliers (Cook’s D < 1), and the model showed good calibration (Hosmer–Lemeshow χ^2^ = 6.42; *p* = 0.38) with moderate explanatory capacity (Nagelkerke R^2^ = 0.21).

Collectively, these findings demonstrate that oral mucosal pathology in incarcerated tobacco users is multifactorial, shaped by behavioral exposure, biological aging, and structural determinants such as confinement duration and poor hygiene practices. This evidence supports the epidemiological need for integrated tobacco-cessation and oral-health promotion interventions within correctional health systems, aligned with national non-communicable disease prevention frameworks.

## 10. Discussion

The results of this study help analyze the tobacco consumption profiles, motivation to quit tobacco, and oral health status among incarcerated people using the TRACE (Tobacco Use, Risk Factors, Assessment, Cessation and Effects through Epidemiology) framework. To the best of our knowledge, this is the first study to examine such trends in the central jail of Navi Mumbai. Tobacco use in correctional facilities remains a significant concern, with reported prevalence rates reaching as high as 92.6% [3]. In this study, 53.1% of inmates reported tobacco use, with 39.5% consuming smokeless forms of tobacco. This disproportionate rate of tobacco use is linked to a variety of factors, including the stressful and restrictive nature of the carceral environment, limited healthcare access, and the social and economic vulnerabilities faced by such individuals.

The study population consisted of 3321 male inmates, with a mean age of 41.76 ± 9.63 years. A substantial proportion (45.1%) were aged between 41 and 50 years, and 45.2% had been incarcerated for 1–5 years. The higher prevalence of tobacco use in this age group could be due to more prolonged exposure to tobacco before incarceration and habitual coping mechanisms formed over time. Oral hygiene practices varied, with 68.1% of inmates using a toothbrush, while 31.9% relied on other methods, indicating a need for health promotion interventions among inmates regarding basic oral hygiene maintenance.

Tobacco use in the form of beedi, cigarettes, snuff, betel quid, and other smokeless forms was observed in 1765 inmates (53.1%), likely driven by factors such as boredom, stress relief, peer influence, or a combination of these. Betel quid was the most common habit (24.0%), followed by cigarette or beedi smoking (13.6%) and Toombak (snuff) use (11.0%). Despite the prohibition of tobacco in Indian jails, the study found a significant number of prisoners engaging in this habit with access to these substances. This highlights potential gaps in law enforcement and prison management by security personnel. These findings align with those of Veera Reddy et al., who reported that 42.8% of prisoners in Karnataka jails had similar adverse tobacco habits [8].

The study also assessed inmates’ readiness to quit tobacco and used the MTSS (Motivation to Stop Scale), adapted from Kotz et al. [9]. Additionally, 76.3% of tobacco users expressed a desire to quit, with 29.5% reporting their intent to stop within three months. However, 82.8% inmates reported the longest time gone without tobacco products as one day only. This discrepancy between motivation and actual cessation highlights a critical window for intervention, suggesting that tailored cessation programs could be highly effective in this setting. However, the availability of such resources remains a concerning barrier, with only a tiny proportion of inmates having access to cessation programs [10].

The prevalence of dental caries in this study was 43.72%, which is relatively lower compared to other studies reporting higher rates of untreated dental conditions [11]. The DMFT index is a widely used method to assess dental caries prevalence and treatment needs. A study in Karnataka’s central prison reported a mean DMFT score of 5.26, indicating a high caries burden [8], which also corroborated with a study among Finnish population reporting a active caries prevalence of greater than 60% [12]. Similarly, a 2022 study in Uttarakhand found a mean DMFT of 5.40 ± 6.49, highlighting poor oral hygiene, limited dental care, and neglect as key contributors to the oral health challenges in incarcerated populations [13]. This study is consistent with others that reported high DMFT scores increasing with age; however, the higher filled teeth index suggests that the sustained efforts of the satellite center located within this central jail may have contributed to reducing the burden of oral health issues among inmates. These findings underscore the need to establish additional satellite dental health centers across all jails to further address oral health disparities.

Periodontal disease was observed in 46.04% of inmates, representing nearly half of the study population and highlighting the compounded impact of tobacco use on oral health. In contrast, previous studies have reported periodontal disease rates as high as 97%, indicating significant variability in prevalence [14].

Oral mucosal lesions (OML) were identified in 86 (4.8%) inmates out of the 1765 tobacco users, with the most prevalent conditions being oral submucous fibrosis (2.3%) and leukoplakia (0.8%). These findings align with a study conducted in Bhopal’s central jail by Arjun et al., which reported similar lesions as the most common, though at a higher prevalence of 34.8% in their population [15].

Our findings underscore the urgent need for tobacco cessation programs tailored to such populations. Without adequate treatment, the vast majority of smokers relapse upon reentry into society, as tobacco use remains prevalent even after release [16]. The risk of relapse remains high due to barriers such as lack of access to healthcare, tobacco retail density in communities, and social stigma.

Several limitations in the study must be acknowledged. These challenges stem from larger, holistic influences and are innate; the mitigation of which needs to be prioritized through organized upstream and downstream approaches.

### 10.1. Underreporting of Smoking in Indian Prisons (Due to Fear of Disciplinary Action)

Tobacco use among incarcerated individuals in India is notably high, with studies indicating that approximately 70–80% of male prisoners are current smokers [3]. However, strict smoking bans in correctional facilities have led to significant underreporting of tobacco use. Inmates often hide their smoking habits because they are afraid of punishment, such as solitary confinement or losing privileges. This underreporting makes it hard to assess how common smoking is and to evaluate the effectiveness of tobacco control policies in prisons. Martin SA et al. studied the effects of smoking bans in English prisons. They found that while these bans aim to reduce tobacco use, they may also cause inmates to underreport their smoking behaviors due to fear of disciplinary action [17]. This issue is pertinent to Indian prisons, where strict enforcement of smoking bans may deter inmates from disclosing their tobacco use, thereby underestimating the true prevalence of smoking within these institutions. The World Health Organization’s report on tobacco use in prisons states that smoking is the most widely used psychoactive substance in these settings, with smoking rates 2 to 4 times higher than in the general population. The report also points out that tobacco use is often underreported because of fear of punishment, particularly in facilities with strict smoking bans [18].

### 10.2. Shared Exposure to Secondhand Smoke

Non-smoking inmates in Indian prisons still face exposure to secondhand smoke, even with smoking bans in place. A study in Bangalore’s Central Jail found that tobacco use is a strong part of prison culture. Factors like boredom and easy access to tobacco products make it harder to stop use completely [3]. This environment increases the risk of secondhand smoke exposure for non-smokers, potentially leading to adverse health outcomes. Studies also characterized the exposure of prison staff to secondhand smoke, elucidating that despite smoking bans, secondhand smoke exposure remained a significant issue, indicating that inmates may still be exposed to secondhand smoke, especially in shared spaces [19]. The WHO report also talks about the high levels of secondhand smoke in prisons. It points out that even with smoke-free cells, non-smoking inmates frequently breathe in secondhand smoke because of poor ventilation and shared areas [18,20].

### 10.3. Legal Framework and Enforcement Challenges

The Supreme Court of India has recognized the detrimental effects of smoking on public health and has issued directives to prohibit smoking in public places, including prisons. For instance, in 2008, the Court upheld the Central Government’s notification to impose a ban on smoking in public places, effective from 2 October 2008 [21]. Additionally, the Model Prison Manual 2016, formulated by the Ministry of Home Affairs, emphasizes the need for uniformity in laws relating to prisons, including provisions for tobacco control [22]. Despite these legal frameworks, enforcement varies widely among states. A study examining compliance with the ban on smoking in public places, including healthcare facilities, found that enforcement was inadequate. This has resulted in continued exposure to tobacco smoke [23]. This inconsistency underscores the need for robust monitoring and adherence to tobacco control policies within correctional institutions.

The reliance on self-reported data for tobacco use may introduce bias, as inmates could underreport consumption due to social desirability. The study’s cross-sectional nature restricts the ability to infer causality between tobacco use and oral health outcomes. A longitudinal study would provide more robust evidence of causality. Moreover, the study exclusively focused on adult male prisoners, thereby limiting the applicability of the findings to female inmates or juveniles. Tobacco use behaviors and oral health outcomes are also influenced by dynamic social and environmental factors, which could lead to variations over time. Lastly, the research was conducted in a single facility with a dental satellite center, restricting the generalizability of the findings to other correctional settings or populations without similar healthcare infrastructure.

## 11. Future Implications

The current psychometric analyses constitute an exploratory validation phase, intended to establish the internal structure and preliminary reliability of TRACE. External validation using independent samples and diverse population datasets remains imperative to confirm its measurement invariance, temporal stability, and generalizability. This foundational testing therefore represents an essential first step toward positioning TRACE as a statistically sound and epidemiologically robust framework for behavioral surveillance, oral–systemic risk assessment, and population health modeling.

Future research should utilize the TRACE (Tobacco Use, Risk Factors, Assessment, Cessation, and Effects through Epidemiological Factors) framework in prisons across India to address the current scarcity of literature in this realm. This framework can guide studies that focus on expanding tobacco cessation programs within correctional facilities, providing comprehensive support through nicotine replacement therapies, counseling, and peer support. Improving access to routine oral healthcare, particularly by establishing dental satellite centers in more prisons, is crucial for addressing the high prevalence of dental caries and periodontal disease. Additionally, enhancing oral health education tailored to the needs of incarcerated individuals can foster better self-care practices and reduce tobacco use. Longitudinal studies based on the TRACE framework are necessary to assess the long-term impact of tobacco cessation and oral health interventions. Integrating such programs into correctional healthcare policies, with a focus on post-release care, will ensure continuity of care and reduce the long-term public health burden.

## 12. Conclusions

This study mapped the oral health and tobacco risk profiles among inmates in a central prison in Navi Mumbai, revealing that 43.7% suffered from dental caries and 46.0% from periodontal disease. More than half (53.1%) of inmates reported tobacco use, and three-quarters of these expressed a willingness to quit—underscoring the urgent need for integrated cessation and oral health programs in correctional settings.

However, as this investigation employed a cross-sectional design, the observed associations between tobacco use and oral health outcomes should be interpreted as correlational rather than causal. Future longitudinal and interventional studies are warranted to confirm temporality, establish causality, and evaluate the impact of targeted cessation and oral health interventions in correctional populations.

Prisons, as part of the Indian Criminal Justice System, must evolve from punitive institutions to centers of rehabilitation, integrating evidence-based oral healthcare and tobacco cessation services. By addressing these disparities, correctional facilities can enhance inmate well-being and mitigate the broader public health burden.

## 13. Ethical Use of Artificial Intelligence Statement

Generative artificial intelligence (AI) tools, including OpenAI’s ChatGPT (GPT-5, OpenAI, San Francisco, CA, USA), were employed exclusively for language refinement and editorial clarity. No AI tools were used for data generation, analysis, or interpretation. All AI-assisted content was thoroughly reviewed, verified, and validated by the authors and domain experts to ensure accuracy, integrity, and compliance with ethical and scientific standards.

## Figures and Tables

**Table 1:** Distribution of prisoners based on demographics.

Variable	*n* (%)
Age group in years	
21–30	298 (9.0%)
31–40	1173 (35.3%)
41–50	1497 (45.1%)
>50	353 (10.6%)
Total	3321 (100%)
Mean ± SD	41.76 ± 9.63
Duration in jail	
<1 year	756 (22.7%)
1–5 years	1502 (45.2%)
5–10 years	922 (27.8%)
>10 years	142 (4.3%)
Total	3321 (100%)
Oral hygiene practices	
Brushing with a toothbrush	2263 (68.1%)
Brushing without a toothbrush	1058 (31.9%)
Total	3321 (100.0%)
Health risk behaviors	
Betel quid	798 (24.0%)
Toombak (snuff)	366 (11.0%)
Others	150 (4.5%)
Cigarette/beedi smoking	451 (13.6%)
Non-tobacco users	1556 (46.9%)
Total	3321 (100.0%)

SD—Standard deviation: All values are expressed as the frequency with percentage (in parentheses).

**Table 2:** Distribution of prisoners based on readiness to quit among tobacco users.

Parameters	N = 1765 (%)
**Longest period of time gone without using tobacco products**	
Less than one day	1462 (82.8%)
Less than one week	237 (13.4%)
Between one week and one month	48 (2.8%)
Between one week and three months	9 (0.5%)
Between three and six months	4 (0.2%)
Twelve months or more	5 (0.3%)
Total	1765 (100.0%)
**Estimated number of previous quit attempts**	
Never	485 (27.5%)
Once	846 (48.0%)
Twice	344 (19.5%)
Between 3 and 5 times	58 (3.2%)
Over 10 attempts	32 (1.8%)
Total	1765 (100.0%)
**Desire to quit**	
Yes	1347 (76.3%)
No	418 (23.7%)
Total	1765 (100.0%)
**MTSS (Motivation To Stop Scale)**	
I do not want to stop using tobacco	418 (23.7%)
I should stop using tobacco, but I do not want to	267 (15.1%)
I want to stop, but I have not thought about when	180 (10.2%)
I really want to stop, but I don’t know when	134 (7.6%)
I want to stop and hope to stop soon	242 (13.7%)
I really want to stop and intend to stop in the <3 months	520 (29.5%)
I really want to stop and intend to stop in <1 month	4 (0.2%)
Total	1765 (100.0%)

All values are expressed as the frequency with percentage (in parentheses).

**Table 3:** Distribution of Decayed, Missing, and Filled Teeth Index and Oral Hygiene Index-Simplified based on different age groups among prisoners.

Age Group in Years (Percentage Population)	DMFT Index				OHI-S Index		
	DT	MT	FT	DMFT	DI-S	CI-S	OHI-S
21–30 (9%)	1.92 ± 0.82	1.01 ± 0.80	2.96 ± 0.18	5.89 ± 1.14	0.99 ± 0.59	0.36 ± 0.15	1.35 ± 0.62
31–40 (35.3%)	2.15 ± 3.54	2.08 ± 1.41	3.80 ± 0.40	8.03 ± 3.85	1.17 ± 0.66	0.81 ± 0.75	1.99 ± 1.22
41–50 (45.1%)	2.02 ± 1.95	3.55 ± 1.71	3.82 ± 0.383	9.39 ± 2.58	1.26 ± 0.65	0.98 ± 0.77	2.24 ± 1.31
>50 (10.6%)	2.04 ± 1.38	5.81 ± 1.66	2.88 ± 0.321	10.73 ± 2.14	1.24 ± 0.64	1.04 ± 0.73	2.28 ± 1.22
*p*-value	<0.001 *	<0.001 *	<0.001 *	<0.001 *	<0.001 *	<0.001 *	<0.001 *

DMFT: Decayed, Missing, Filled teeth; DT: Decayed Teeth; MT: Missing Teeth; FT: Filled Teeth; OHI-S: Oral Hygiene Index-Simplified; DI-S: Debris Index- Simplified; CI-S: Calculus Index-Simplified; All values are expressed as Mean ± standard deviation (SD). The statistical test used: ANOVA test; level of significance: * *p* ≤ 0.001 is considered statistically significant.

**Table 4:** Association between demographic variables and Decayed Missing Filled Teeth (DMFT) Index/Oral Hygiene Index-Simplified (OHI-S) Index among prisoners.

Parameters	Coefficient r	SE	t	95% CI	*p*-Value	Adjusted R^2^
**Dependent variable: Decayed Missing Filled Teeth (DMFT) Index**						
Constant	4.446	0.214	21.155	4.034–4.858	<0.001 *	0.151
Age	1.540	0.065	23.575	1.412–1.668	<0.001 *	
Oral hygiene practices	0.585	0.111	5.270	0.367–0.802	<0.001 *	
Health risk behavior	−0.127	0.104	−1.219	−0.332–0.77	0.223	
**Dependent variable: Oral Hygiene Index-Simplified (OHI-S) Index**						
Constant	1.280	0.087	14.714	1.109–1.450	<0.001 *	0.037
Age	0.285	0.027	10.548	0.232–0.338	<0.001 *	
Oral hygiene practices	0.149	0.046	3.248	0.059–0.239	<0.001 *	
Health risk behavior	−0.062	0.043	−1.443	−0.147–0.022	0.149	

CI: Confidence interval; SE: Standard error; The statistical test used: Multivariate linear regression model; Level of significance: * *p* ≤ 0.001 is considered highly statistically significant.

**Table 5:** Prevalence and Predictors of Oral Mucosal Lesions (OML) Among Tobacco Users (N = 1765).

Predictor Variable	OML Prevalence *n* (%)	Adjusted OR (aOR)	95% CI for aOR	*p*-Value
Lesion Type (Prevalence)				
Oral submucous fibrosis	42 (2.3)	–	–	–
Leukoplakia	15 (0.8)	–	–	–
Lichen planus	7 (0.3)	–	–	–
Ulceration	10 (0.5)	–	–	–
Candidiasis	12 (0.6)	–	–	–
Any OML (overall)	86 (4.8)	–	–	–
Risk Factors (Logistic Regression)				
Age > 40 years	–	1.72	1.12–2.65	0.014 *
Incarceration ≥ 5 years	–	1.49	1.01–2.21	0.046 *
Smokeless tobacco use	–	2.38	1.56–3.64	<0.001 **
Dual use (smoked + smokeless)	–	2.11	1.14–3.90	0.017 *
Poor oral hygiene (no toothbrush)	–	1.81	1.23–2.67	0.002 **
DMFT (per unit increase)	–	1.05	1.01–1.08	0.006 **
OHI-S (per unit increase)	–	1.11	1.02–1.21	0.012 *
Model Diagnostics				
Hosmer–Lemeshow χ^2^ (p)			6.42 (0.38)	–
Nagelkerke R^2^			0.21	–
Mean VIF (range)			1.46 (1.02–1.91)	–
Tolerance (range)			0.52–0.98	–
Box–Tidwell linearity test (*p*)			>0.05 (non-significant)	–
Cook’s distance (max)			<1.0 (no influential outliers)	–

Note: OML = Oral Mucosal Lesion; OR = Odds Ratio; CI = Confidence Interval; DMFT = Decayed, Missing, Filled Teeth; OHI-S = Oral Hygiene Index-Simplified. * *p* < 0.05, ** *p* < 0.01. Multicollinearity was evaluated using Variance Inflation Factor (VIF) and tolerance statistics (VIF < 2.0, tolerance > 0.50 for all predictors), indicating no collinearity. The model demonstrated good fit (Hosmer–Lemeshow *p* = 0.38) and moderate explanatory strength (Nagelkerke R^2^ = 0.21).

## Data Availability

The datasets generated and/or analyzed during the current study are not publicly available due to ethical and legal restrictions associated with research on incarcerated individuals. Data may be made available from the corresponding author upon reasonable request and with prior approval from the Institutional Ethics Committee and the relevant prison administration.
